# A novel approach to quantifying individual's biological aging using Korea’s national health screening program toward precision public health

**DOI:** 10.1007/s11357-024-01079-2

**Published:** 2024-02-02

**Authors:** Jinho Yoo, Junguk Hur, Jintae Yoo, Donald Jurivich, Kyung Ju Lee

**Affiliations:** 1 YooJin BioSoft, 24, Jeongbalsan-Ro Ilsandong-Gu, Goyang-Si Gyeonggi-Do, 10402 Korea; 2 Department of Biomedical Sciences, School of Medicine and Health Sciences, University of North Dakota, Grand Forks, ND 58202 USA; 3 Department of Geriatrics, School of Medicine and Health Sciences, University of North Dakota, Grand Forks, ND 58202 USA; 4 Department of Women’s Rehabilitation, National Rehabilitation Center, 58, Samgaksan-Ro, Gangbuk-Gu, Seoul, 01022 Korea; 5 Institute for Occupational & Environmental Health, Korea University, Seoul, 02841 Korea

**Keywords:** Biological Aging, Differential Aging and Health Index, Mortality Prediction, Sex-Specific Aging

## Abstract

Accurate prediction of biological age can inform public health measures to extend healthy lifespans and reduce chronic conditions. Multiple theoretical models and methods have been developed; however, their applicability and accuracy are still not extensive. Here, we report Differential Aging and Health Index (DAnHI), a novel measure of age deviation, developed using physical and serum biomarkers from four million individuals in Korea's National Health Screening Program. Participants were grouped into aging statuses (< 26 vs. ≥ 26, < 27 vs. ≥ 27, …, < 75 vs. ≥ 75 years) as response variables in a binary logistic regression model with thirteen biomarkers as independent variables. DAnHI for each individual was calculated as the weighted mean of their relative probabilities of being classified into each older age status, based on model ages ranging from 26 to 75. DAnHI in our large study population showed a steady increase with the increase in age and was positively associated with death after adjusting for chronological age. However, the effect size of DAnHI on the risk of death varied according to the age group and sex. The hazard ratio was highest in the 50–59-year age group and then decreased as the individuals aged. This study demonstrates that routine health check-up biomarkers can be integrated into a quantitative measure for predicting aging-related health status and death via appropriate statistical models and methodology. Our DAnHI-based results suggest that the same level of aging-related health status does not indicate the same degree of risk for death.

**Supplementary Information:**

The online version contains supplementary material available at 10.1007/s11357-024-01079-2.

## Introduction

The World Health Organization periodically releases healthy lifespan indicators, such as life expectancy, mortality, and disability-adjusted life year, to optimize health for everyone [1]. However, these metrics may not resonate at the individual level as they provide information based on the average population whose living conditions are heavily affected by factors such as sex, age, race, ethnicity, or disability. The increase in morbidity and mortality during the recent COVID-19 pandemic has sharpened the focus on individual healthy lifestyle plans within the public healthcare system [2–4] as a means to reduce mental and physical burdens, improve immunological functions, and prevent chronic diseases and disability towards increased life expectancy.

Currently, over 74% of global deaths are attributable to non-communicable diseases, including chronic conditions [5]. Age stands as the most prevalent risk factor for these diseases, contributing significantly to the overall disease burden [6, 7]. With aging-related issues steadily increasing, accurately quantifying aging is critical for developing preventive interventions and therapies for aging-related diseases. This effort also involves enhancing personal health strategies, which is a key component in public health initiatives [2, 4], aimed at promoting healthy aging, reducing disease burden, and enhancing the quality of life for individuals.

Predicting biological age is essential for estimating mortality and life expectancy in population health management. From the moment of birth, individuals sharing the same chronological age may exhibit different levels of biological aging. The concept of biological age, an individual’s age defined by the degree of biological changes at cellular and molecular levels, is ascertained through a variety of well-standardized biomarkers pertinent to health functions [8]. These biomarkers offer a more precise depiction of an individual’s physiological or functional age. Biological age is considered an important informative marker, highlighting variations in human organ systems compared to chronological age [8–10]. Yet, the relationship between biological age, organ systems, chronological age, and age-related diseases continues to be a subject of extensive research. Aging biomarkers serve as crucial tools for identifying and evaluating biological age. Through the Biomarkers of Aging Consortium (https://www.agingconsortium.org), a consensus on different aspects of aging biomarkers has been reached [8]. The comprehensive approach of the Consortium includes establishing consensus on key terminology, classifying biomarkers from a regulatory perspective, elucidating use cases based on existing biomarkers and trials, and assessing biomarkers, for instance, using validated geroprotectors.

Estimating the biological age for differentiating health status involves various theoretical models and methods. Approaches such as multiple linear regression (MLR) analysis [11–15], principal component analysis (PCA) [16, 17], Hochschild’s method [18–20], and Klemera and Doubal’s method [21], along with machine learning techniques, including stochastic gradient descent (SGD) [22], deep neural network (DNN) [23, 24], and random forest (RF) [23, 25], have been widely used for this purpose. However, previous computational methods for estimating biological age had limitations, such as restricted biomarker accessibility, model feasibility, limited sample representativeness, an arbitrary transformation of unitless values to unit year, and over- or under-estimation of biological ages in young and old age groups, respectively [18, 26–28]. Notably, in many studies, biological ages tend to be higher than chronological ages among deceased individuals, emphasizing the significance of health and suggesting a more robust association of biological ages or biochemical parameters with mortality [17, 29].

It is imperative to have a comprehensive understanding of biological age and its correlation to health for the enhanced prediction of mortality risks, better health status assessment, and improvement of overall health outcomes. Therefore, we have taken measures to address the weaknesses identified in our prior validation of biological age as a predictor of mortality risk [30–33]. This study aimed to enhance risk assessment accuracy by developing a complementary measure to chronological age, utilizing readily available biomarkers from national health screenings. For this purpose, we utilized the Korean National Health Insurance Service (NHIS) database, which offers a wealth of health screening data at a national level. Using these data, we developed a novel tool called the Differential Aging and Health Index (DAnHI) and evaluated its effectiveness in predicting mortality profiles.

## Methods

### Study population and data cleansing

The study population consisted of individuals randomly selected from the Korean NHIS database (NHIS-2020–1-344), which comprises four million individuals (two million males and two million females). General health screening data collected in 2009 were used for the analysis. Dates and causes of death, if any, until 2019, were obtained from Statistics Korea. The study design was retrospective and approved by the Korea University Institutional Review Board (ID: IRB-2019–0271).

Thirteen health screening parameters were collected, including body mass index (BMI), waist circumference (WST), systolic blood pressure (SBP), diastolic blood pressure (DBP), fasting blood sugar (FBS), high-density lipoprotein (HDL), low-density lipoprotein (LDL), triglyceride (TG), hemoglobin (HGB), aspartate aminotransferase (AST), alanine aminotransferase (ALT), and gamma-glutamyl transpeptidase (GGTP), and creatinine (CR).

Participants meeting the following criteria were excluded from the study: (1) age below 20 years; (2) presence of chronic diseases, such as hypertension, diabetes mellitus, cancer, or stroke in the data collection year; (3) death due to extrinsic causes, such as suicide, accident, infectious disease, pregnancy-related death, or unknown causes; (4) health screening parameter outliers determined as < 0.05%, > 99.95%, or missing value. Each of the two study cohorts, male and female, was randomly split into two independent datasets at a 7:3 ratio. The larger dataset served as the development dataset for constructing prediction models, whereas the smaller dataset was used for performance assessment.

For sensitivity analysis, the subgroups were defined based on sex, age range, and cause of death. The death events were further classified into cancer death, non-cancer death, and all-cause death categories. The cancer death group included only deaths attributed specifically to cancer, encompassing individuals who succumbed to various forms of malignancies, such as lung cancer, breast cancer, colorectal cancer, and other types of cancer. The non-cancer death group encompassed all deaths, excluding cancers and external causes, such as suicide, accidents, infectious diseases, pregnancy-related death, or cases with unknown reasons. The all-cause death group included both cancer and non-cancer deaths, representing the entire spectrum of mortality within the study population.

### Constructing a statistical model to quantify aging-related health status

A statistical model was constructed using a multivariable binary logistic regression approach to quantify aging-related health status. The chronological age was repeatedly dichotomized to define 50 separate aging statuses, such as < 26 vs. ≥ 26 years, < 27 vs. ≥ 27 years, and so on, up to < 75 vs. ≥ 75 years. Health screening parameters were utilized as independent variables in the logistic regression models. Based on these models, an aging-related health status indicator known as the Differential Aging and Health Index (DAnHI) was computed for each participant. The calculation procedure for DAnHI is summarized as follows:

- **Step 1**: Set n = 26 (minimum model age)
- **Step 2**: Define the dichotomous aging status for each model age, n

0_n_: persons with age < n

1_n_: persons with age ≥ n

- **Step 3**: Construct a multivariable binary logistic regression model and compute P_i,n_ (= ${\text{p}}\left({{\text{Y}}}_{{\text{i}}}={1}_{{\text{n}}}\right)$in Eq. 1)
- **Step 4**: Compute C_n_ from the values of P_i,n_ using a receiver operating characteristic
  **(**ROC) curve analysis to maximize Youden’s J statistic
- **Step 5**: Repeat Step 2 – Step 5 for n = 27, 28 … and 75
- **Step 6**: Compute DAnHI_i_ (Eq. 2)

Eq. 1: Individual’s probability to be differentiated as ≥ n years:

$${\text{p}}\left({\text{Y}}=\mathrm{1}_{{\text{n}}}\right)=\frac{{\mathrm{exp}}\left({\sum }_{{\text{k}}=0}^{{\text{p}}}{\beta }_{{\text{k}}}{{\text{X}}}_{{\text{k}}}\right)}{\mathrm1{\mathrm+}\,{\mathrm{exp}}\left({\sum }_{{\text{k}}=0}^{{\text{p}}}{\beta }_{{\text{k}}}{{\text{X}}}_{{\text{k}}}\right)}$$

$$\text{p}\left({\mathrm{Y}}_\text{i}=\mathrm{1}_\text{n}\right)\mathrm:\;\mathrm{the}\;\mathrm{probability}\;\mathrm{of}\;\mathrm{i}^\text{th}\;\mathrm{individual}\;\mathrm{to}\;\mathrm{be}\;\mathrm{differentiated}\;\mathrm{as}\;\geq\;\mathrm{n}\;\mathrm{years}\;\mathrm{group}$$

$$\mathrm{Y}_{\mathrm i}:\mathrm i^{\mathrm{th}}\;\mathrm{individual}'\mathrm s\;\mathrm{aging}\;\mathrm{status}$$

$$\text{i}=1,2,\dots,\mathrm{and}\;N\;\left(\text{N}:\mathrm{total}\;\mathrm{number}\;\mathrm{of}\;\mathrm{study}\;\mathrm{participants}\right)$$

$$\text{n}=26,27,\dots,\mathrm{and}\;75\;\left(\mathrm{model}\;\mathrm{age}\;\mathrm{observed}\;\mathrm{in}\;\mathrm{the}\;\mathrm{development}\;\mathrm{data}\right)$$

$${\text{X}}_\text{k}:\text{k}^\text{th}\;\mathrm{independent}\;\mathrm{variable}\;\left(\mathrm{health}\;\mathrm{screening}\;\mathrm{parameter}\right)$$

$${\beta }_{{\text{k}}}:\mathrm{regression}\;\mathrm{coefficient}\;\mathrm{of}\;{{\text{k}}}^{{\text{th}}}\;\mathrm{independent}\;\mathrm{variable}\left({\beta}_{0}\;\mathrm{means}\;\mathrm{intercept}\right)$$

$${\text{p}:\mathrm{total}\;\mathrm{number}\;\mathrm{of}\;\mathrm{independent}\;\mathrm{variable}}$$

Eq. 2: Individual’s differential aging and health index (DAnHI_i_):

$${DAnHI}_{i}=\frac{{\sum }_{n=26}^{75}n\times {\delta }_{i,n}}{75-26+1}$$

$${\delta }_{i,n}={P}_{i,n}-{C}_{n}$$

$$\text{i}=1,2,\dots,\mathrm{and}\;N\;\left(\text{N}:\mathrm{total}\;\mathrm{number}\;\mathrm{of}\;\mathrm{the}\;\mathrm{study}\;\mathrm{population}\right)$$

$${DAnHI}_{i}:\;\mathrm{weighted}\;\mathrm{mean}\;\mathrm{of}\;{\delta }_{i,n}$$

$${\text{C}}_\text{n}:\;\mathrm{cutoff}\;\mathrm{of}\;{\text{P}}_{\text{i},\text{n}}\;\mathrm{determined}\;\mathrm{at}\;\mathrm{the}\;\mathrm{point}\;\mathrm{that}\;\mathrm{maximizes}\;\mathrm{Youden}'s\;\mathrm J\;\mathrm{statistic}\;\mathrm{in}\;\mathrm{the}\;\mathrm{ROC}\;\mathrm{curve}\;\mathrm{analysis}$$

### Evaluation of the predictive performance of DAnHI for mortality

The independent effect of DAnHI on mortality was estimated as a hazard ratio (HR) using the chronological age as an adjusting covariate in the multivariable Cox proportional hazards regression model. In addition, the HR (%) was calculated as (HR of DAnHI–1.0)/(HR of chronological age –1.0) × 100 (%), to evaluate the relative influence size of DAnHI on mortality compared with chronological age. Furthermore, the study participants were grouped into three subgroups according to the size of DAnHI: the low-risk (DAnHI ≤ 0), medium-risk (0 < DAnHI < median of positive DAnHIs), and high-risk (DAnHI ≥ median of positive DAnHIs) groups. The survival probabilities of the three risk groups over time were compared against each other using Kaplan–Meier curves and a log-rank test.

Model performance of DAnHI for predicting mortality was assessed in terms of the area under the curve (AUC) during the follow-up periods of up to 10 years using the validation datasets. Four Cox proportional hazards regression models were generated using DAnHI, chronological age, chronological age with DAnHI, and BA (biological age, defined by chronological age + DAnHI) as the independent risk factors. The risk scores, calculated from the Cox models, were used to compute AUCs over time, and the difference of AUCs between the DAnHI-including models and the chronological age-only model was analyzed using DeLong’s method. In addition, the death prediction performance was compared between chronological age and biological ages that were estimated using DAnHI and other statistical or machine learning-based algorithms, such as MLR, PCA, SGD, DNN, and RF.

### Data handling and statistical analysis

The data handling for the Korean cohort data from the NHIS database was performed using SAS (SAS v9.4, Cary, NC, USA). All statistical analyses, including data-cleansing, construction of the multivariable binary logistic regression models, Kaplan–Meier curve analysis with log-rank test, Cox proportional hazards regression analysis, and time-dependent ROC curve analyses with DeLong’s test, were performed using R v4.0.3 (R Foundation for Statistical Computing, Vienna, Austria) and T&F program v4.0 (YooJinBioSoft, Seoul, Korea).

## Results

### Characteristics of the nationwide study cohorts

Figure S1 illustrates the selection process of the study participants. Four million individuals who participated in the Korea National General Health Screening Programs in 2009 were randomly selected from the NHIS database. After removing participants meeting the exclusion criteria, the final study cohort consisted of 3,125,936 participants, comprising 1,579,322 males and 1,546,614 females. Table S1 and Fig. S2 illustrate the overall distribution of the study cohorts’ demographic and health screening parameters.

**Fig. 1:**
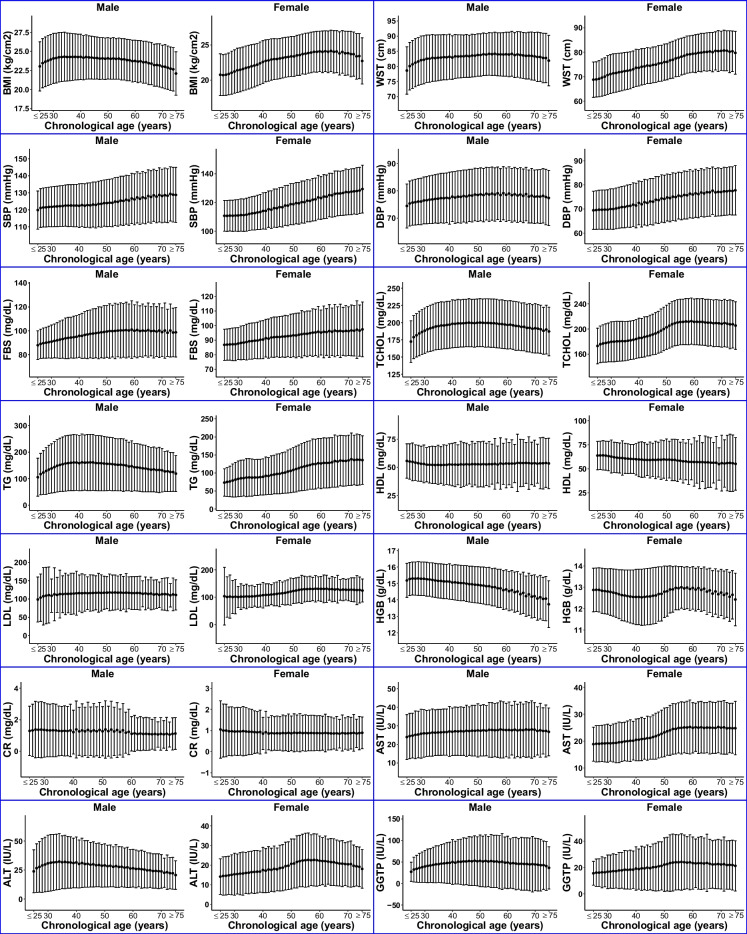
Overall distribution of the health screening parameters. Chronological age-related patterns of the health screening parameters are represented as mean ± standard deviation

Figure 1 provides a comprehensive view of the distribution of the health screening parameters, highlighting their diverse patterns across chronological ages and sex. These metrics exhibit heterogeneous trends, with some showing continuous increases and others showing fluctuations or both increases and subsequent decreases over time. Notably, sex differences are evident in several parameters. For instance, the TG levels increase until the late thirties or early forties and then slowly decrease in male participants. In contrast, a monotonically increasing trend with increasing chronological age was observed in female participants.

Moreover, SBP consistently rises as the study participants get older, irrespective of sex. However, other parameters display varying degrees of sex-specific differences. BMI, for example, demonstrates an initial increase followed by a subsequent decrease at around mid-thirties and mid-sixties in male and female participants, respectively. The increasing pattern of WST is more prevalent in females compared with males. In addition, the HGB levels in males show a continuous decrease over time, whereas those in females exhibit fluctuating patterns.

### Independent impact of health screening parameters on age-specific differentiation

**Fig. 2:**
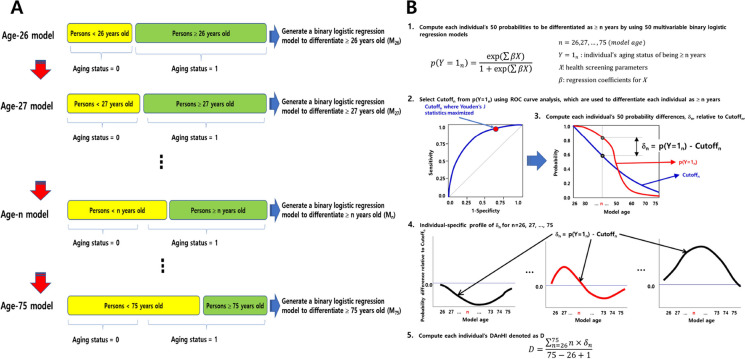
Procedures to compute DAnHI. (**A**) Fifty multivariable binary logistic regression models were constructed to differentiate ages ≥ 26, ≥ 27, …, and ≥ 75. Panel (**B**) illustrates the overall process of calculating DAnHI for each participant. Τhe three distinctive patterns of δ_n_ computed for each study participant are illustrated in step #4. In the middle pattern, the *D* sums up near 0 since the areas above and below Y axis = 0 counterbalance overall. *D* becomes a large negative value as in the left-side pattern and a positive value in the right-side pattern

To investigate the independent impact of the health screening parameters on age-specific differentiation, a novel approach was employed utilizing 50 multivariable binary logistic regression models (Fig. 2A). Table 1 presents the significant effects of all parameters in differentiating participants aged ≥ 40 years from those aged < 40 years (P-values < 2.37 × 10^–12^) as well as participants aged ≥ 50 years from those aged < 50 years (P-values < 1.71 × 10^–34^). The ages of 40 and 50 years were selected as important cutoff points for age differentiation based on prior research and clinical relevance [34–36]. Most parameters exhibited the same direction of effect in differentiating participants aged ≥ 40 years from those aged < 40 years, regardless of sex, except for BMI and DBAn increase in BMI was negatively associated with participants aged ≥ 40 years (odds ratio = 0.834; 95% CI: 0.832–0.836) in males but positively associated with participants aged ≥ 40 years (odds ratio = 1.053; 95% CI: 1.051–1.056) in females. A positive or negative association indicates that the increment of the parameter acts as an agonist or antagonist in differentiating more-aged such as those ≥ 40 years compared with < 40 years. Conversely, DBP showed a completely opposite pattern to BMI in differentiating aged ≥ 40 years (odds ratio = 1.015 for males; odds ratio = 0.994 for females).

**Table 1:** The independent effects of health screening parameters on differentiating ≥ 40 years and ≥ 50 years

	Model for differentiating ≥ 40 years				Model for differentiating ≥ 50 years			
Health screening parameters	Male		Female		Male		Female	
	OR (95%CIs)	P-value	OR (95%CIs)	P-value	OR (95%CIs)	P-value	OR (95%CIs)	P-value
BMI (kg/cm^2^)	0.834 (0.832–0.836)	< 1.00 × 10^–310^	1.053 (1.051–1.056)	< 1.00 × 10^–310^	0.794 (0.792–0.797)	< 1.00 × 10^–310^	0.909 (0.907–0.911)	< 1.00 × 10^–310^
WST (cm)	1.092 (1.091–1.093)	< 1.00 × 10^–310^	1.060 (1.059–1.061)	< 1.00 × 10^–310^	1.112 (1.111–1.114)	< 1.00 × 10^–310^	1.093 (1.092–1.094)	< 1.00 × 10^–310^
SBP (mmHg)	1.003 (1.003–1.004)	1.05 × 10^–44^	1.030 (1.029–1.031)	< 1.00 × 10^–310^	1.024 (1.023–1.024)	< 1.00 × 10^–310^	1.037 (1.037–1.038)	< 1.00 × 10^–310^
DBP (mmHg)	1.015 (1.014–1.015)	< 1.00 × 10^–310^	0.994 (0.993–0.995)	3.61 × 10^–47^	0.995 (0.995–0.996)	6.75 × 10^–38^	0.986 (0.985–0.986)	< 1.00 × 10^–310^
FBS (mg/dL)	1.022 (1.022–1.023)	< 1.00 × 10^–310^	1.022 (1.021–1.022)	< 1.00 × 10^–310^	1.015 (1.014–1.015)	< 1.00 × 10^–310^	1.010 (1.009–1.010)	< 1.00 × 10^–310^
TCHOL (mg/dL)	–	–	–	–	–	–	–	–
TG (mg/dL)	1.001 (1.000–1.001)	5.04 × 10^–108^	1.005 (1.005–1.005)	< 1.00 × 10^–310^	0.999 (0.999–0.999)	3.18 × 10^–114^	1.005 (1.005–1.005)	< 1.00 × 10^–310^
HDL (mg/dL)	0.996 (0.996–0.996)	1.61 × 10^–216^	0.996 (0.996–0.996)	2.02 × 10^–232^	0.996 (0.996–0.996)	1.30 × 10^–174^	0.998 (0.997–0.998)	3.64 × 10^–94^
LDL (mg/dL)	1.004 (1.004–1.004)	< 1.00 × 10^–310^	1.008 (1.008–1.009)	< 1.00 × 10^–310^	1.002 (1.002–1.002)	< 1.00 × 10^–310^	1.009 (1.008–1.009)	< 1.00 × 10^–310^
HGB (g/dL)	0.705 (0.702–0.708)	< 1.00 × 10^–310^	0.871 (0.867–0.874)	< 1.00 × 10^–310^	0.675 (0.673–0.678)	< 1.00 × 10^–310^	1.067 (1.062–1.071)	1.16 × 10^–229^
CR (mg/dL)	0.981 (0.979–0.984)	3.34 × 10^–49^	0.915 (0.911–0.920)	5.76 × 10^–307^	0.968 (0.965–0.971)	2.38 × 10^–94^	0.959 (0.954–0.963)	2.08 × 10^–61^
AST (IU/L)	1.038 (1.038–1.039)	< 1.00 × 10^–310^	1.077 (1.076–1.078)	< 1.00 × 10^–310^	1.039 (1.039–1.040)	< 1.00 × 10^–310^	1.084 (1.083–1.085)	< 1.00 × 10^–310^
ALT (IU/L)	0.972 (0.971–0.972)	< 1.00 × 10^–310^	0.988 (0.987–0.988)	2.93 × 10^–180^	0.968 (0.967–0.968)	< 1.00 × 10^–310^	0.974 (0.973–0.975)	< 1.00 × 10^–310^
GGTP (IU/L)	1.004 (1.004–1.004)	< 1.00 × 10^–310^	1.001 (1.001–1.002)	2.37 × 10^–12^	1.001 (1.001–1.001)	4.18 × 10^–63^	0.998 (0.998–0.998)	1.71 × 10^–34^

The male cohorts included 485,359 (43.9%; < 40 years), 620,166 (56.1%; ≥ 40 years), 783,676 (70.9%; < 50 years), and 321,849 (29.1%; ≥ 50 years) participants, while the female cohorts included 329,831 (30.5%; < 40 years), 752,799 (69.5%; ≥ 40 years), 670,112 (61.9%; < 50 years), and 412,518 (38.1%; ≥ 50 years) participants. TCHOL was excluded from the multivariable binary logistic regression model due to multicollinearity with TG, HDL, and LDL. Variance inflation factor (VIF) < 3.0 was used as a cutoff for multicollinearity

Abbreviations: ALT: alanine aminotransferase, AST: aspartate aminotransferase, BMI: body mass index, CI: confidence intervals, CR: creatinine.., DBP: diastolic blood pressure, FBS: fasting blood sugar, GGTP: gamma-glutamyl transpeptidase, HDL: high-density lipoprotein, HGB: hemoglobin, LDL: low-density lipoprotein, OR: odds ratio, SBP: systolic blood pressure, TCHOL: total cholesterol, TG: triglyceride, WST: waist circumference

The patterns of effect direction differed between the comparison of participants aged ≥ 50 years and those aged < 50 years, and the comparison of participants aged ≥ 40 years and those aged < 40 years, in several parameters, such as BMI, DBP, TG, HGB, and GGTFor example, TG showed a negative association in male participants but a positive association in female participants (odds ratio = 0.999 for male TG; odds ratio = 1.005 for female TG), whereas HGB showed a negative association in male participants but a positive association in female participants (odds ratio = 0.675 for male HGB; odds ratio = 1.067 for female HGB). In contrast, GGTP showed a positive association in male participants but a negative association in female participants (odds ratio = 1.001 for males; odds ratio = 0.998 for females). When differentiating ≥ 50 years, the association pattern of DBP and TG in males, as well as BMI and GGTP in females, reversed from positive to negative compared with differentiating aged ≥ 40 years. Moreover, the association pattern of HGB reversed from negative to positive, which was observed exclusively in females. These age-specific patterns emphasize the importance of considering age differences in the interpretation of health screening parameters and their associations with biological ages, while accounting for potential variations based on sex.

The impact of each health screening parameter on differentiating a specific age is compared in Fig. S3. For instance, the GGTP serum levels revealed the largest odds ratio in the Age-26 model in males, suggesting that this value exerts the biggest positive influence on differentiating male participants with ≥ 26 years compared with the other parameters. The GGTP effect gradually declined as the model age increased to 52–53 years and then reversed. In contrast, a physiological metric, the SBP effect, changed its effect direction from negative to positive around the mid- to late-thirties and exhibited a gradual increase in positive effect as the mode age increased. In females, the serum GGTP levels did not show a substantial change across all model ages, whereas SBP consistently showed increasing positive effects as the model age increased. Overall, the effect size and direction of health screening parameters show sex-specific differences and change with increasing model age.

### Differential Aging and Health Index (DAnHI)

This study developed a novel measure — the differential aging and health index (DAnHI) — using mandatory nationwide health screening parameters to properly represent aging-related differential health status for individuals with different health conditions. Figure 2B illustrates the overall process of computing each individual’s DAnHI based on the 50 multivariable binary logistic regression models. For example, if δ_n_ for all model ages is > 0, *D* is always calculated as > 0, indicating an “increased aging health status.” However, if δ_n_ for all model ages is < 0, *D* is always calculated as < 0, indicating a “reduced aging health status.” If the individual profile has both positive and negative values across the model ages, then *D* ≈0 suggests that the individual is under “stable aging health status.”

**Fig. 3:**
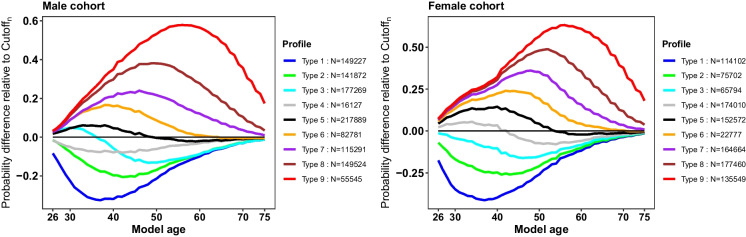
Distinctive profile types of δ_n_. The probability of each individual being ≥ *n* years old, p(*Y* = 1_n_), was computed from the model M_n,_ as illustrated in Fig. 2. The Cutoff_n_ value was selected at the point where Youden’s *J* statistics was maximized using the receiver operating characteristic (ROC) curve analysis. The differences between p(*Y* = 1_n_) and Cutoff_n_ were calculated as δ_n,_ indicating a model age-specific profile of the differential aging of each individual. K-means clustering analysis with nine random centroids was performed, and the thick profile lines indicate the averages of participants in each clustered grouDAnHI: differential aging and health index

Figure 3 illustrates several distinctive profile patterns of δ_n_, generated by K-means clustering with nine random centroids. These profiles could be primarily grouped into three distinct patterns (Fig. 2B; Step 4), where δ_n_ – Cutoff_n_ = 0 serves as the indicator of an antagonist and agonist role in an individual’s “differential aging health status.” This equation helps identify three distinct profile patterns (shown in the left, middle, and right panels in Fig. 2B; Step 4). Individuals with a profile pattern in the middle panel have DAnHI ≈0, suggesting that the aging-related health status is stable or not different from individuals of the same age. In contrast, individuals whose profile patterns fall into the left or right panels can be interpreted as being relatively healthier or unhealthier, considering their age.

**Fig. 4:**
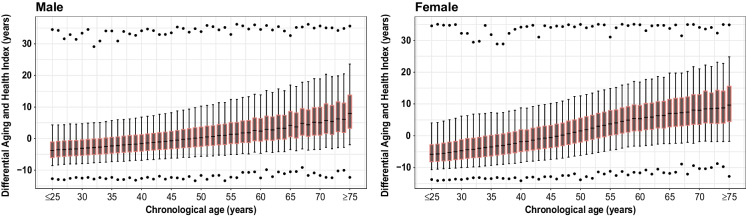
Age-specific distribution of DAnHI. Male and female cohorts are presented in box plots with medians (solid horizontal lines) and interquartile ranges (IQR) of DAnHI computed at each chronological age. Outer whiskers indicate 5% and 95% of DAnHI, and the maximum and minimum values are presented as black dots in the lower and upper parts of the graphs. The gradual increase pattern of DAnHI and wider IQR is observed as the participants get older for all the cohorts. DAnHI: differential aging and health index

Age-specific distributions of DAnHI for males and females are illustrated in Fig. 4. DAnHI gradually increased as the participants aged, which suggests that the aging-related health status deteriorates faster in older individuals. A wider range of DAnHI observed in the older participants suggests that person-to-person variance dominates as individuals age. Figure S4 illustrates diverse age- and sex-specific correlations between DAnHI and health screening parameters. For instance, the pattern of TG shows considerable fluctuation across different age groups in males. It is negatively correlated prior to the age of 40 years, then exhibits a positive correlation from the ages of 40 to 60 years, only to revert to a negative correlation after 60 years. In contrast, regardless of age, TG consistently presents positive correlations in females.

### Age- and sex-specific association between DAnHI and mortality

The independent effect of DAnHI on the risk of death was evaluated as an HR in a multivariable Cox proportional hazards regression model after adjusting for the effect of chronological age. Mortality data from Statistics Korea were analyzed, covering a follow-up period of up to 10 years from the time of data collection in the study cohorts (Table S2).

The average follow-up period was 9.3 years (range 0.0–9.9 years). Table S3 shows that both DAnHI and chronological age have significant but distinct independent effects on mortality risk across various age groups and both sexes. For example, in males aged 40–49 years, a one-year increase in DAnHI was associated with a 9.7% increase in total mortality (HR = 1.097, 95% confidence interval [CI] = 1.091–1.104), while a one-year increase in chronological age corresponded to a 10.5% increase in total mortality (HR = 1.105, 95% CI = 1.092–1.119). In contrast, among the oldest participants (≥ 70 years), the impact of DAnHI on the total mortality decreased to 3.5% (HR = 1.035, 95% CI = 1.032–1.037), whereas the chronological age increased total mortality by 25.1% (HR = 1.251, 95% CI = 1.239–1.263), which was more than double the effect observed in males aged 40–49 years. Among females, both DAnHI and chronological age had weaker effects than that in males for the same 40–49-year-old participants (HR = 1.041 for DAnHI; HR = 1.050 for chronological age). The impact of DAnHI decreased more than chronological age among participants aged ≥ 70 years (HR = 1.008 for DAnHI; HR = 1.424 for chronological age). Moreover, the effect of DAnHI was more pronounced for non-cancer deaths compared with cancer deaths in both male and female participants aged 40–49 years (HR = 1.131 vs. 1.067 for males; HR = 1.085 vs. 1.026 for females).

**Fig. 5:**
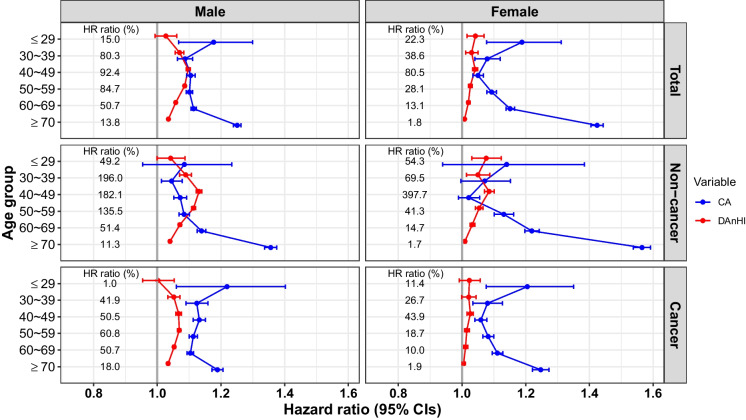
Hazard ratios for DAnHI relative to chronological age according to age group, sex, and mortality type. Any deaths due to extrinsic causes, such as suicide, accident, infectious disease, pregnancy-related death, or unknown causes (IDC10 codes: A00-B99, O00-O99, Q00-Q99, R95-R99, S00-T98, and V01-Y98) were excluded from the current study. Total: all-cause death, Non-cancer: death by non-cancer, Cancer: death by cancer (ICD10 codes: C00-C97 and D00-D48), CA: chronological age, DAnHI: differential aging health index, HR: hazard ratio, CIs: confidence intervals. HR ratio (%) = (HR for DAnHI–1.0) / (HR for CA–1.0) × 100 (%)

Notably, the HRs for DAnHI show an opposite trend to chronological age as individuals age. The HR ratio (%), which represents the ratio of the two HRs (DAnHI relative to chronological age), steadily increases until the age of 40–49 or 50–59 years, followed by a decline (Fig. 5**)**. For example, considering total mortality in male participants, the HR ratio (%) increased from 15.0% in the 20 s to 80.3% in the 30 s and 92.4% in the 40 s, followed by a declining pattern of 84.7% in the 50 s, 50.7% in the 60 s, and 13.8% in the 70 s. Although DAnHI tends to be higher in older age groups compared with the other age groups (Fig. 4), its impact on mortality is marginal.

**Fig. 6:**
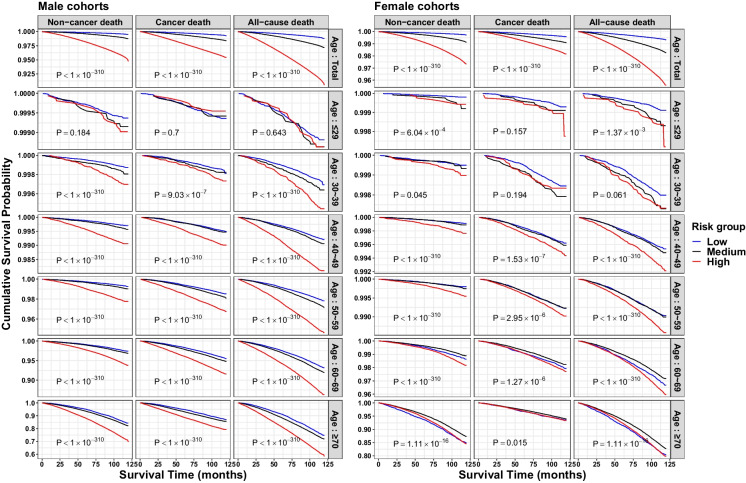
Comparison of the survival curves for the three risk groups across age group, sex, and mortality type. The survival probabilities of the three risk groups over time were compared against each other using Kaplan–Meier curves and a log-rank test. The Y-axis ranges were wider for the older age groups. Abbreviations: DAnHI: differential aging and health index, Total: all age groups, P: P-value from log-rank test. It should be noted that the scales in Y-axes are consistent only at the same age groups within each cohort

The smallest HR ratio (%) was observed in the oldest age group (≥ 70 years), except for the male cohort with cancer-caused death. The larger confidence intervals of HR for DAnHI in the youngest age group (≤ 29 years) were mainly due to minimal numbers of death. The pattern of HR ratio (%) is more pronounced in the male cohort and non-cancer deaths. To further assess the risk of high DAnHI on death, the participants were classified into three risk groups based on DAnHI: low-risk (DAnHI ≤ 0), medium-risk (0 < DAnHI < median of positive DAnHIs), and high-risk (DAnHI ≥ median of positive DAnHIs) groups. Survival probabilities for the three risk groups, determined by DAnHI magnitude, were compared using Kaplan–Meier curves with a log-rank test, revealing significant differences in most age groups, except for the youngest age group (log-rank test *p values* < 9.03 × 10^–7^, Fig. 6).

In addition to assessing the independent effect of DAnHI on mortality, we also investigated its predictive capability using the AUC for follow-up periods of up to 10 years. We constructed four Cox proportional hazards regression models, utilizing DAnHI, chronological age, chronological age with DAnHI, and biological age (= chronological age + DAnHI) as independent risk factors. Table 2 summarizes and compares the discrimination accuracies of these models in predicting non-cancer deaths using the validation dataset with broad range groups (Table S4). In most age groups, the “chronological age with DAnHI” models demonstrated improved performances compared with the chronological age-only models. However, for individuals aged ≥ 65 years, the predictive performances of the biological age models were lower compared with those of the chronological age-only models (0.715 vs. 0.739 in males and 0.665 vs. 0.779 in females, respectively). Detailed analysis results of prediction accuracies for other types of mortality can be found in Tables S5 and S6.

**Table 2:** Prediction accuracy of the DAnHI-including models for predicting 10-year non-cancer death

Sex	Age range	Statistics	DAnHI	CA	CA with DAnHI	BA
Male	Total	AUC (95% CIs)	0.803 (0.797–0.809)	0.891 (0.886–0.895)	0.900 (0.896–0.905)	0.901 (0.897–0.905)
		DeLong's P	8.85 × 10^–181^	Ref	7.75 × 10^–63^	1.02 × 10^–30^
	≤ 39	AUC (95% CIs)	0.612 (0.575–0.649)	0.626 (0.592–0.660)	0.657 (0.622–0.691)	0.657 (0.622–0.692)
		DeLong's P	0.554	Ref	0.018	0.016
	40–64	AUC (95% CIs)	0.698 (0.686–0.711)	0.690 (0.679–0.702)	0.751 (0.740–0.762)	0.750 (0.739–0.761)
		DeLong's P	0.322	Ref	8.35 × 10^–40^	2.64 × 10^–50^
	≥ 65	AUC (95% CIs)	0.638 (0.628–0.648)	0.739 (0.730–0.748)	0.757 (0.749–0.766)	0.715 (0.706–0.724)
		DeLong's P	4.28 × 10^–57^	Ref	1.02 × 10^–25^	5.91 × 10^–08^
Female	Total	AUC (95% CIs)	0.786 (0.779–0.793)	0.927 (0.923–0.932)	0.928 (0.923–0.932)	0.917 (0.912–0.921)
		DeLong's P	< 1.00 × 10^–310^	Ref	8.53 × 10^–07^	1.71 10^–29^
	≤ 39	AUC (95% CIs)	0.688 (0.613–0.762)	0.749 (0.689–0.809)	0.780 (0.723–0.836)	0.777 (0.718–0.835)
		DeLong's P	0.172	Ref	0.083	0.285
	40–64	AUC (95% CIs)	0.648 (0.628–0.668)	0.713 (0.693–0.732)	0.725 (0.706–0.744)	0.720 (0.701–0.738)
		DeLong's P	7.21 × 10^–08^	Ref	2.46 × 10^–04^	0.217
	≥ 65	AUC (95% CIs)	0.558 (0.546–0.569)	0.779 (0.771–0.787)	0.782 (0.774–0.791)	0.665 (0.655–0.675
		DeLong's P	4.10 × 10^–214^	Ref	2.17 × 10^–04^	2.11 × 10^–106^

The prediction evaluation was done separated in each male and female cohort. DAnHI: cox-model with differential aging and health index as a single risk factor. CA: cox-model with chronological age as a single risk factor. CA with DAnHI: cox-model with CA and DAnHI as complementary risk factors. BA: cox-model with biological age, defined as the sum of CA and DAnHI, as a single risk factor. AUC (95% CIs): area under the curve with 95% confidence intervals. DeLong's P: P-value computed by DeLong's method to compare AUCs between models

Abbreviations: AUC: area under the curve, BA: biological age, CA: chronological age, Ref.: reference, DAnHI: differential aging and health index

The comparison of 10-year mortality prediction accuracy between chronological age and biological ages, estimated using various machine learning-based and statistical algorithms, including DAnHI, is summarized in Tables S7 and S8. In the male cohort, the highest prediction performance was observed with DAnHI (AUC = 0.830), followed by chronological age (AUC = 0.823) for total mortality prediction. Other algorithms demonstrated lower prediction performances (AUCs < 0.8). Notably, for all biological age estimation algorithms, as well as chronological age, discrimination accuracy was higher in predicting non-cancer deaths than cancer-related deaths. DAnHI consistently showed higher discrimination accuracy across all mortality types (Table S7). Conversely, in the female cohort, chronological age yielded a slightly higher AUC (0.842) for total mortality prediction compared to DAnHI (AUC = 0.833), with all other algorithms exhibiting lower accuracy (Table S8). Additionally, the female cohort demonstrated more precise prediction for non-cancer deaths compared to cancer-related deaths than the male cohort.

## Discussion

In this study, we developed a novel index called the Differential Aging and Health Index (DAnHI) to accurately estimate the biological aging status of an individual using readily accessible biomarkers from a health screening program. We demonstrated that the distribution of health screening parameters varied with chronological age and sex, and the age and sex-specific correlations between these parameters and DAnHI provided insights into the associations between aging-related health factors and biomarkers commonly used in health screenings. Unlike other models that directly estimate biological age from statistical or machine learning models [13, 17, 22–25], our model first calculated DAnHI, an independent factor representing differential aging, or age deviation. Subsequently, it incorporated this factor along with the chronological age to enhance accuracy in estimating the risk of death. Lastly, we also found that there is a non-linear relationship between DAnHI and mortality, which suggests that the impact of DAnHI on mortality risk may change with the individual’s age and sex, highlighting the dynamic nature of biological aging.

Various studies [11–21] have been conducted since the early 2000s to predict aging and life expectancy and identify aging-predictive biomarkers. The American Federation on Aging Research (https://www.afar.org/) and the Biomarkers of Aging Consortium (https://www.agingconsortium.org) released various panels of healthy-aging biomarkers, encompassing molecular biomarkers derived from specific molecules and omics, physiological biomarkers based on functional performance and physical characteristics, as well as digital biomarkers measured through wearable and non-wearable technologies [8, 32]. However, most of these biomarkers are not available in measurement, general and handling at the population level. To overcome this difficulty in accessing healthy-aging biomarkers, we used large-scale national health screening data, which are readily accessible and representative of the general population. By leveraging this comprehensive dataset, we constructed models incorporating these biomarkers, aggregated into DAnHI, to assess their impact on the health of an individual. Our approach is similar to that employed by national insurance groups such as the British National Health Service and the United States Centers for Medicare & Medicaid Services in using health data to inform healthcare services and policies [37].

In the Korea National General Health Screening Programs data, 13 health parameters were identified as easily measurable, popular, and computable anthropometric and molecular biomarkers. Derived from these parameters, DAnHI effectively predicts age deviation from chronological age. While big data and machine learning methods for biological age prediction, as advocated by the Biomarkers of Aging Consortium, have expanded assessment criteria, they often function as 'black box' systems, obscuring the underlying predictive mechanisms. In contrast, the DAnHI model not only demonstrates feasibility and validity but also offers a more interpretable approach, enhancing understanding of each parameter's contribution. This distinction leads to clearer results interpretation and potentially more effective healthcare interventions. Moreover, DAnHI reveals individual variations in aging according to chronological age, providing a novel perspective in aging research.

Our DAnHI model addresses the limitations associated with previous methods in predicting aging or biological age. These limitations include unitless values in PCA (Principal Component Analysis) models, the tendency of MLR (Multiple Linear Regression) models to overestimate or underestimate biological age in young and older adults, and the 'black box' nature of machine learning algorithms that obscures their internal decision-making processes. In response to these challenges, our model adopts a distinct approach by acknowledging the complex interactions between each screening parameter and chronological age. Instead of solely focusing on potential linear associations like previous models, our DAnHI model highlights the intricate, age-specific impacts of each parameter, revealing their dynamic interplay. This approach has unveiled a nuanced connection between DAnHI and mortality, characterized by varying patterns across different ages. This finding suggests that the impact of DAnHI on mortality risk is not static but evolves as individuals age, reflecting the intricate interplay between aging-related biomarkers and the aging process itself. As individuals navigate through different stages of life, the influence of DAnHI on mortality becomes more pronounced, suggesting the evolving and complex nature of biological aging.

In the current study, we observed that the impact of biomarkers, as aggregated into DAnHI, exhibited an overall increasing pattern with wider ranges as the chronological age increased. This finding suggests that the influence of these biomarkers on aging becomes more substantial in older generations, with greater person-to-person variations observed in both males and females. Specifically, we found that the turning point of the impact of DAnHI on aging, indicated by the age when the median DAnHI becomes zero, was between the ages of 48 and 50 years in males and between the ages of 45 and 47 years in females. These observations indicate the significant and progressive influence of biomarkers on aging as individuals grow older and experience the effects of aging more profoundly.

It should be noted that DAnHI is a quantitative measure that represents the differential aging of an individual based on 50 binary logistic regression models (Fig. 2A). DAnHI can be directly interpreted as the gap between the estimated biological age and the chronological age of each individual. This characteristic distinguishes our model from previous approaches that predict biological age first and then compute the age gap by subtracting chronological age from the predicted biological age. Our model directly captures the differential aging status and provides a more accurate estimation of the aging trajectory of an individual, accounting for the interplay of multiple factors contributing to the aging process.

DAnHI, independently of chronological age, was highly predictive of death within 10 years. A more pronounced predictability was observed in men than in women, and in non-cancer-related deaths than in cancer-related deaths (Fig. 5). Furthermore, the integration of DAnHI and chronological age enhanced the accuracy of death prediction in both males and females (Table 2). The higher predictability for non-cancer-related deaths may be due to the nature of the 13 health parameters selected for the DAnHI model, which primarily focus on metabolic and cardiovascular health. These biomarkers were chosen for their availability in routine health screenings and their relevance in aging research. Consequently, DAnHI might be inherently more sensitive to detecting biological age variations related to non-cancerous conditions. This predictive capability highlights the necessity for age- and sex-specific aging prediction models, as our age and sex-stratified analysis unveiled distinct patterns. These patterns emphasize the potential benefits of tailoring predictions to align with specific age and sex characteristics.

The majority of health screening parameters explored in this study, including those related to glucose and lipid metabolism, serve as widely utilized health biomarkers. However, they often lack age-specific referencing values for adults aged 20 or 30 years and older. For example, as shown in Fig. 1, biomarkers related to glucose and lipid metabolism showed variable mean change patterns across different age groups and sex that were potentially associated with hormonal change. In Figure S4, these biomarkers display varying correlations with DAnHI across different age groups and sexes. These observations suggest the complexity of biological aging and highlight the necessity for a nuanced understanding of its process. The interpretation of these dynamic patterns, potentially reflective of underlying hormonal inflences, will require further investigation.

It has long been recognized that there are differences between the sexes when it comes to energy metabolism [34, 38]. The emergence of metabolic syndrome, attributed directly to the influence of sex hormones during menopause [39, 40], provides compelling evidence that glucose and lipid metabolism undergo direct regulation by estrogen and testosterone. Either estrogen deficiency or a relative increase in testosterone is implicated in inducing insulin resistance, leading to an atherosclerotic lipid profile [40, 41]. Moreover, there is notable evidence that the prevalence of hypertension rises more rapidly among older women than among older men [42, 43].

Sex differences notably impact the pathophysiology of aging, particularly in the expression of biomarkers associated with aging and age-related diseases, including those related to the gonadal and adrenal endocrine systems, as well as immune function [44]. Notably, we discovered variations in the age at which significant changes occur in these biomarkers, highlighting the pivotal role of sex-specific analysis in aging research. These turning points represent moments of homeostatic adjustment, reflecting distinct adaptive responses to aging in men and women. This phenomenon illustrates the concept of 'adaptive homeostasis,' where the homeostatic range for multiple functions transiently expands or contracts, adapting to different physiological states without initiating a repair process [35]. When these biomarkers return to their normal ranges, the adaptive responses cease, indicating a dynamic and fluctuating process of aging. This underscores the necessity for further research to investigate age-specific marker patterns and their implications in different age groups, enhancing our understanding of sex-specific aging processes. As a detailed exploration of these patterns is beyond the scope of this study, further research is warranted to investigate age-specific marker patterns and their implications. Future studies should explore normative ranges, average values, and the extent of marker changes related to diseases in different age groups to gain a comprehensive understanding of sex-specific aging processes.

To enable a more comprehensive and longitudinal investigation into future DAnHI variations and predictability, it is essential to enhance the DAnHI model involves integrating proposed additional parameters. This enhancement aligns with the biomarker framework outlined in the United States Food and Drug Administration’s Biomarkers, EndpointS, and other Tools resources (FDA-BEST) [45], considering racial groups. This entails incorporating molecular biomarkers, physiological biomarkers including psycho-social health parameters and factors such as smoking, and digital biomarkers, paving the way for a thorough exploration of DAnHI variability and predictability. These additions will allow for a more comprehensive longitudinal examination of DAnHI variation and its predictability. Ultimately, integrating a DAnHI-based biological age prediction model into an individual's health management plan could help identify preventable risk factors for age-related diseases. Furthermore, DAnHI has the potential to improve both public and individual health and social services by facilitating person-centered healthcare and aging prevention, potentially reducing medical expenses at both individual and national levels.

## Conclusions

In conclusion, this study developed the novel DAnHI as an accurate estimator of the biological aging status of an individual using readily accessible biomarkers. DAnHI demonstrates high predictability for mortality and can identify varying levels of mortality risk across sex and age subgroups. Therefore, DAnHI holds significant potential to enhance the accuracy of mortality risk prediction and promote individualized health planning.

### Supplementary Information

Below is the link to the electronic supplementary material.

Supplementary file1 (DOCX 2032 KB)

Supplementary file2 (DOCX 92 KB)

## Data Availability

Restrictions apply to the availability of these data. Data were obtained from the Korean National Health Insurance Sharing Service (NHISS) and are available at https://nhiss.nhis.or.kr with the permission of the NHISS.

## Abbreviations

AFAR: American Federation on Aging Research
ALT: Alanine aminotransferase
AST: Aspartate aminotransferase
AUC: Area under the curve
BA: Biological age
BMI: Body mass index
CI: Confidence interval
CR: Creatinine
DAnHI: Differential Aging and Health Index
DBP: Diastolic blood pressure
DNN: Deep neural network
FBS: Fasting blood sugar
GGTP: Gamma-glutamyl transpeptidase
HDL: High-density lipoprotein
HGB: Hemoglobin
HR: Hazard ratio
LDL: Low-density lipoprotein
MLR: Multiple linear regression
NHIS: National Health Insurance Service
PCA: Principal component analysis
RF: Random forest
ROC: Receiver operating characteristic
SBP: Systolic blood pressure
SGD: Stochastic gradient descent
TG: Triglyceride
WST: Waist circumference
