# Supplementary material for: A novel approach to quantifying individual's biological aging using Korea’s national health screening program toward precision public health
Source: GeroScience. 2024 Feb 2;46(3):3387–403. doi: 10.1007/s11357-024-01079-2 (PMC11009216; doi:10.1007/s11357-024-01079-2)
Supplement: Supplementary file 1 — Supplementary file1 (DOCX 2032 KB) [file 11357_2024_1079_MOESM1_ESM.docx]

Supplementary Materials for

**A novel approach to quantifying individual's biological aging using Korea’s national health screening program toward precision public health**

**This file includes:**

Figure S1. Selection of study participants.

Figure S2. Distribution of the standardized means of health screening parameters.

Figure S3. Odds ratios of the health screening parameters included in each age-differentiating model.

Figure S4. Age-specific correlation between DAnHI and the health screening parameters.

Table S1. Participant characteristics.

Table S2. Summary of mortality in the development and validation data set separated by the cause of death, sex, and decade-specific age groups.

Table S3. Independent effects of chronological age and DAnHI on the risk of mortality.

Table S4. Summary of mortality in the development and validation data set separated by the cause of death, sex, and broad age groups.

Table S5. Prediction accuracy of the DAnHI-including models for predicting 10-year total mortality.

Table S6. Prediction accuracy of the DAnHI-including models for predicting 10-year cancer death.

Table S7. Comparison of prediction performance between chronological age and other biological age estimation algorithms in male cohort.

Table S8. Comparison of prediction performance between chronological age and other biological age estimation algorithms in female cohort.

**
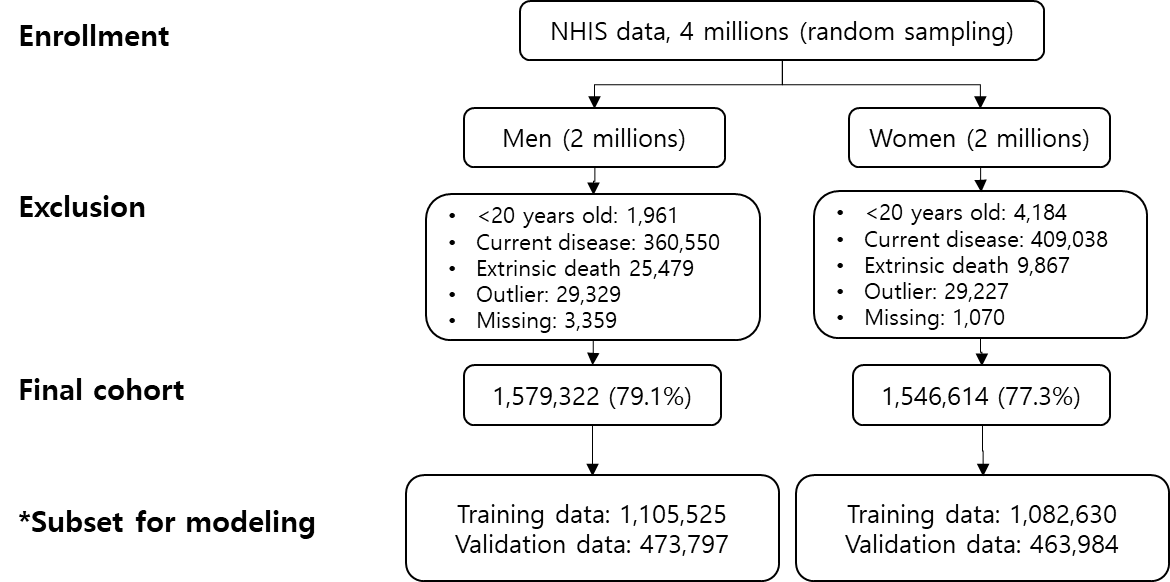
**

**Figure S1. Selection of study participants.**

An outlier was defined as ≤ 0.05% or ≥ 99.95% values for each parameter. NHIS: National Health Insurance Service. *Subset for modeling: randomly split into two independent data sets at a ratio of 7:3.

**
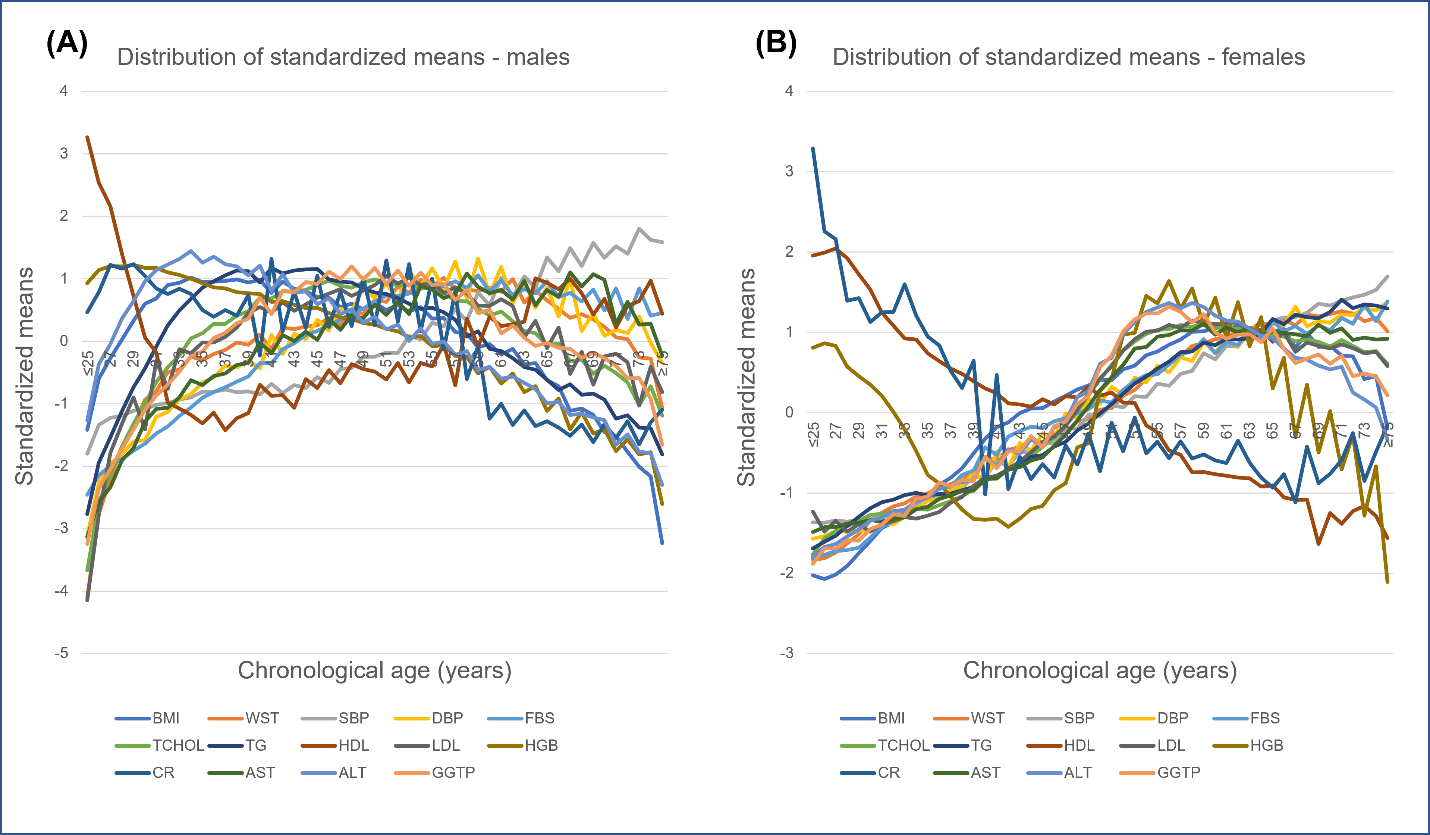
**

**Figure S2. Distribution of the standardized means of health screening parameters**.

The means of each age group were standardized across different ages for each parameter within males (**A**) and females (**B**), respectively.

Abbreviations: BMI: body mass index, SBP: systolic blood pressure, DBP: diastolic blood pressure, FBS: fasting blood sugar, TCHOL: total cholesterol, HGB: hemoglobin, AST: aspartate aminotransferase, ALT: alanine aminotransferase, GGTP: gamma-glutamyl transpeptidase, WST: waist circumference, HDL: high-density lipoprotein, LDL: low-density lipoprotein, TG: triglyceride, CR: creatinine.

(A) Male


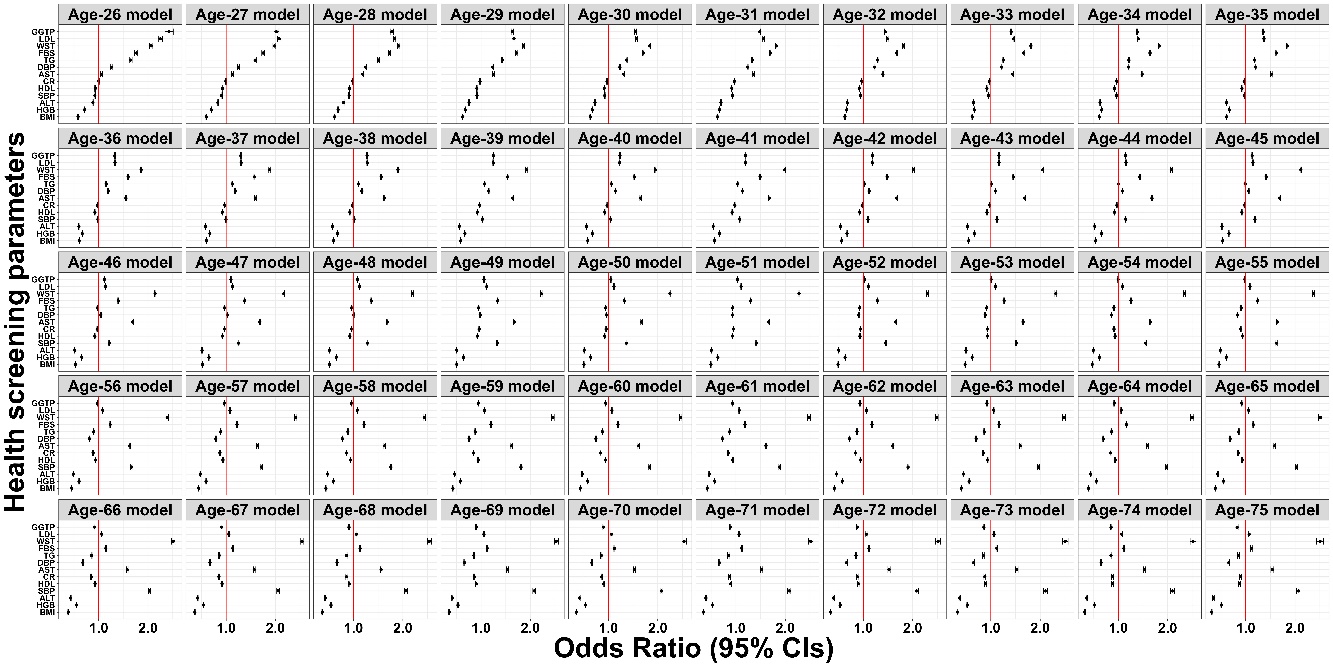


(B) Female


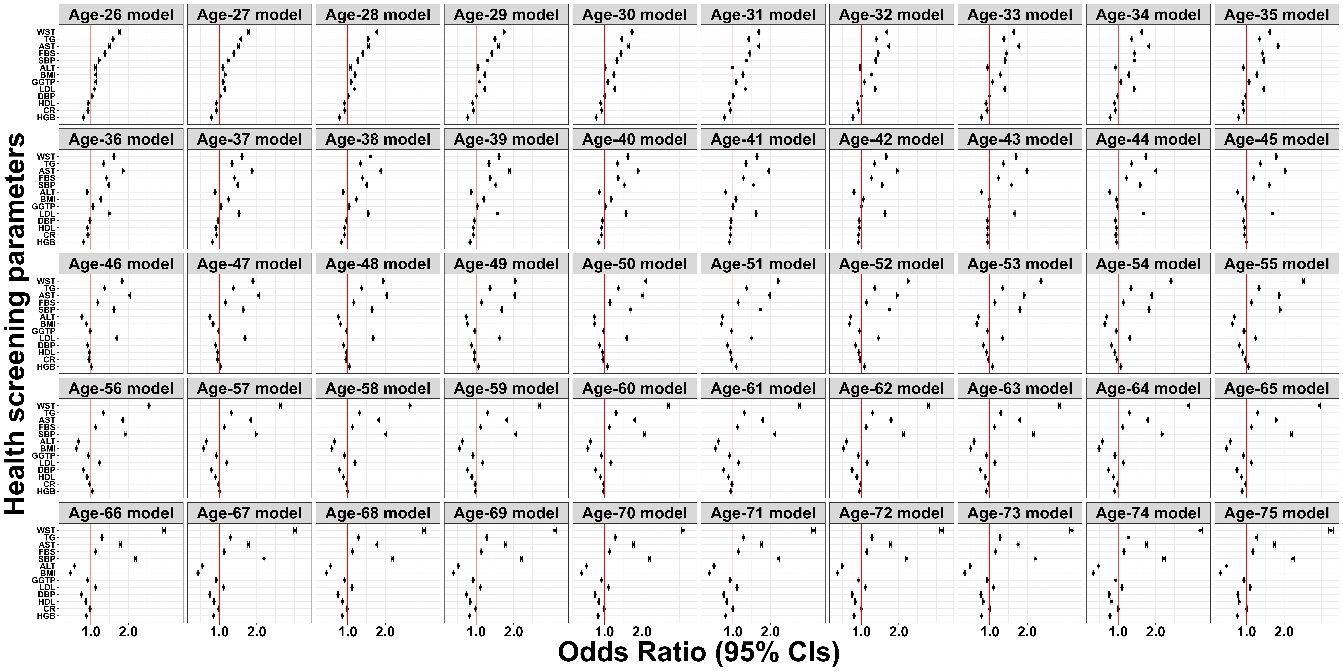


**Figure S3. Odds ratios of the health screening parameters included in each age-differentiating model.**

Age-26 model, …, Age-75 models: multivariable binary logistic regression model to differentiate age ≥ 26, …, ≥75, respectively, in the male and female cohorts. Health screening parameters are sorted by ascending order of odds ratio in the Age-26 model. The vertical red line indicates the odds ratio = 1.0, which represents the point where the parameter’s effect direction changes from negative to positive in each age-differentiating model. The odds ratios were standardized as the standard deviation unit change in each parameter, considering the different units of each parameter.

(A) Male


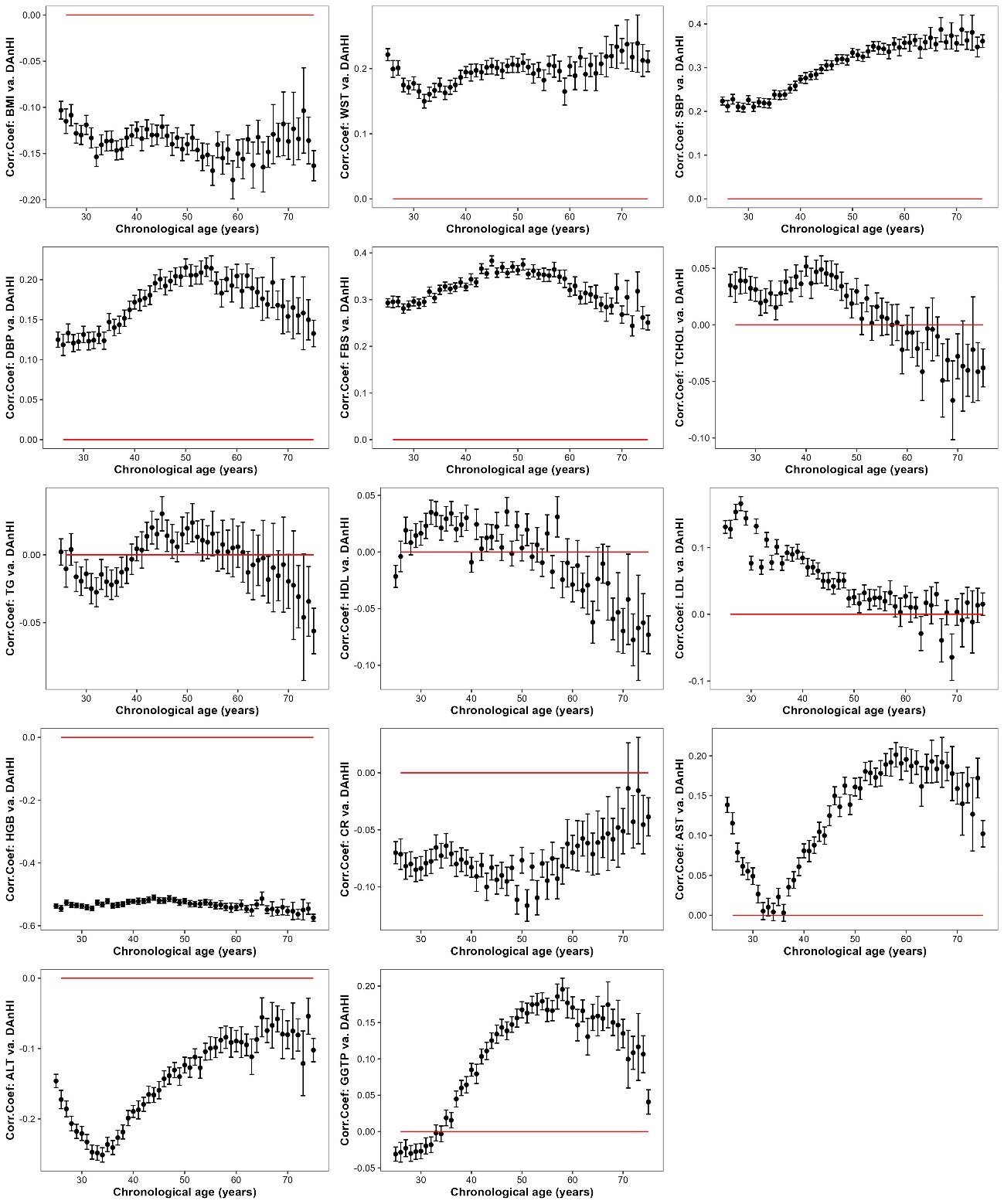


(B) Female


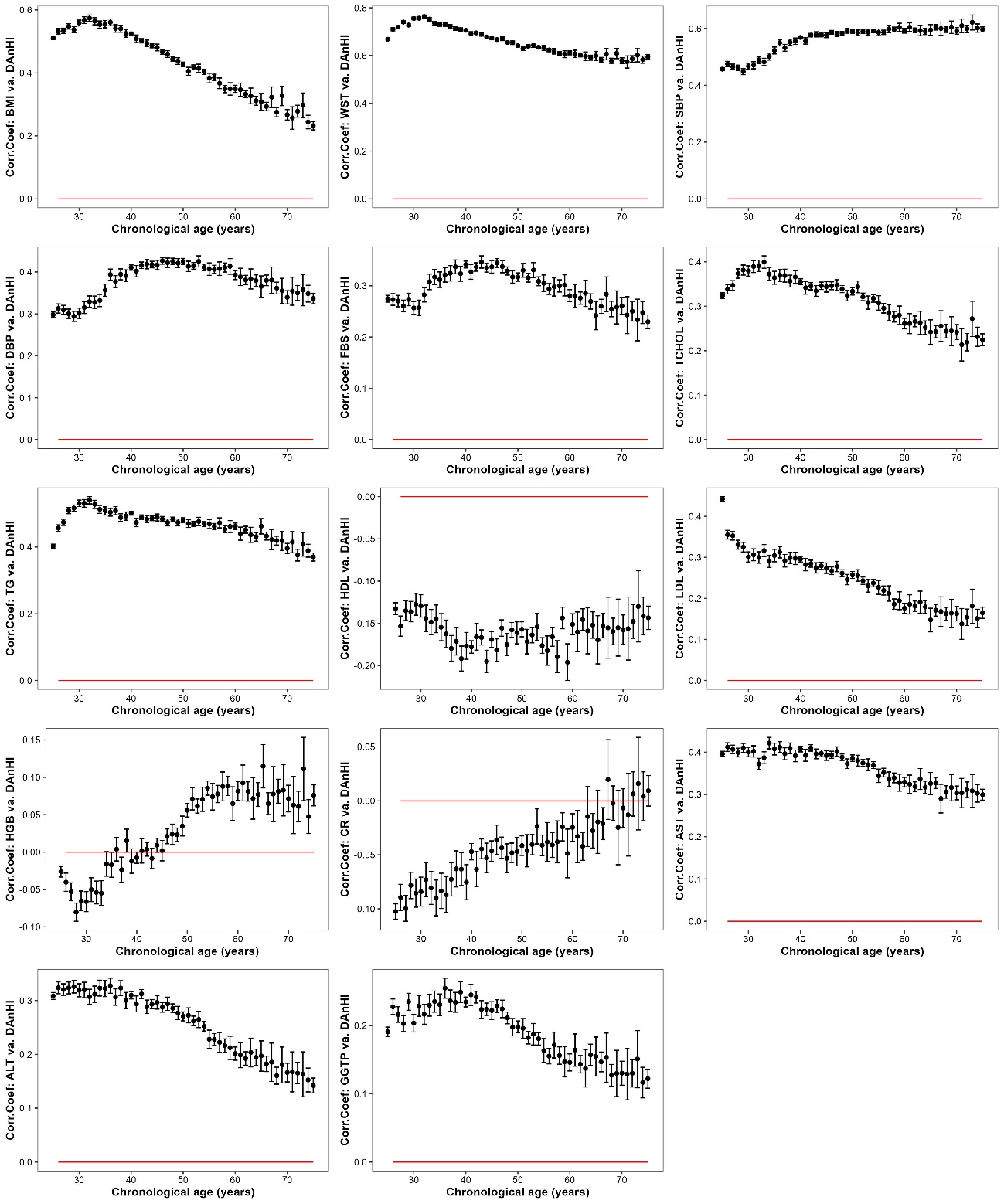


**Figure S4. Age-specific correlation between DAnHI and the health screening parameters**.

The Pearson correlation coefficients and 95% confidence intervals between DAnHI and each health screening parameter at each age group are illustrated for the (**A**) male and (**B**) female cohorts. The red horizontal line in each panel indicates correlation coefficient = 0.

DAnHI: differential aging and health index

**Supplementary Tables**

**Table S1.** **Participant characteristics**

| **Parameters** | **Male** | **Female** | **P value** |
| --- | --- | --- | --- |
|  | **(n=1,579,322)** | **(n=1,546,614)** |  |
| Age (years) | 43.2 ± 12.4 | 45.5 ± 13.1 | <1.0 x 10^-310^ |
| BMI (kg/cm^2^) | 24.0 ± 3.0 | 22.7 ± 3.1 | <1.0 x 10^-310^ |
| WST (cm) | 82.8 ± 7.6 | 74.7 ± 8.3 | <1.0 x 10^-310^ |
| SBP (mmHg) | 123.3 ± 13.3 | 117.2 ± 14.4 | <1.0 x 10^-310^ |
| DBP (mmHg) | 77.4 ± 9.3 | 73.0 ± 9.6 | <1.0 x 10^-310^ |
| FBS (mg/dL) | 95.7 ± 19.1 | 91.9 ± 14.3 | <1.0 x 10^-310^ |
| TCHOL (mg/dL) | 194.2 ± 34.8 | 193.9 ± 36.1 | 5.85 x 10^-15^ |
| TG (mg/dL) | 148.1 ± 98.3 | 102.1 ± 60.6 | <1.0 x 10^-310^ |
| HDL (mg/dL) | 53.0 ± 18.4 | 59.7 ± 20.0 | <1.0 x 10^-310^ |
| LDL (mg/dL) | 113.8 ± 54.1 | 115.7 ± 54.4 | 7.82 x 10^-210^ |
| HGB (g/dL) | 15.0 ± 1.1 | 12.8 ± 1.2 | <1.0 x 10^-310^ |
| CR (mg/dL) | 1.3 ± 1.6 | 0.9 ± 1.0 | <1.0 x 10^-310^ |
| AST (IU/L) | 26.7 ± 13.3 | 21.9 ± 8.6 | <1.0 x 10^-310^ |
| ALT (IU/L) | 29.1 ± 20.6 | 18.6 ± 11.9 | <1.0 x 10^-310^ |
| GGTP (IU/L) | 45.9 ± 50.6 | 20.2 ± 17.4 | <1.0 x 10^-310^ |
|  |  |  |  |

Parameters are presented as mean ± standard deviation. P values were computed using an independent two-sample T-test between male and female.

Abbreviations: BMI: body mass index, SBP: systolic blood pressure, DBP: diastolic blood pressure, FBS: fasting blood sugar, TCHOL: total cholesterol, HGB: hemoglobin, AST: aspartate aminotransferase, ALT: alanine aminotransferase, GGTP: gamma-glutamyl transpeptidase, WST: waist circumference, HDL: high-density lipoprotein, LDL: low-density lipoprotein, TG: triglyceride, CR: creatinine.

**Table S2. Summary of mortality in the development and validation data set separated by the cause of death, sex, and decade-specific age groups.**

| Age group | Type | Development, M | Development, F | Validation, M | Validation, F |
| --- | --- | --- | --- | --- | --- |
| ≤29 | Total participant | 157,337 | 175,426 | 67,152 | 74,665 |
|  | All death | 187 | 169 | 75 | 56 |
|  | Non-cancer | 105 | 44 | 41 | 8 |
|  | Cancer | 82 | 125 | 34 | 48 |
| 30–39 | Total participant | 328,022 | 154,405 | 141,235 | 65,917 |
|  | All death | 998 | 327 | 421 | 161 |
|  | Non-cancer | 488 | 85 | 218 | 46 |
|  | Cancer | 510 | 242 | 203 | 115 |
| 40–49 | Total participant | 298,317 | 340,281 | 127,283 | 146,622 |
|  | All death | 2,977 | 1,743 | 1,337 | 702 |
|  | Non-cancer | 1,298 | 406 | 589 | 167 |
|  | Cancer | 1,679 | 1,337 | 748 | 535 |
| 50–59 | Total participant | 187,755 | 244,171 | 80,639 | 104,744 |
|  | All death | 5,748 | 2,646 | 2,523 | 1,140 |
|  | Non-cancer | 2,143 | 695 | 931 | 322 |
|  | Cancer | 3,605 | 1,951 | 1,592 | 818 |
| 60–69 | Total participant | 93,583 | 115,783 | 40,032 | 49,336 |
|  | All death | 8,583 | 3,700 | 3,693 | 1,649 |
|  | Non-cancer | 3,373 | 1,536 | 1,462 | 676 |
|  | Cancer | 5,210 | 2,164 | 2,231 | 973 |
| ≥70 | Total participant | 40,511 | 52,564 | 17,456 | 22,700 |
|  | All death | 12,773 | 9,393 | 5,431 | 4,083 |
|  | Non-cancer | 7,409 | 6,666 | 3,204 | 2,897 |
|  | Cancer | 5,364 | 2,727 | 2,227 | 1,186 |
| Total | Total participant | 1,105,525 | 1,082,630 | 473,797 | 463,984 |
|  | All death | 31,266 | 17,978 | 13,480 | 7,791 |
|  | Non-cancer | 14,816 | 9,432 | 6,445 | 4,116 |
|  | Cancer | 16,450 | 8,546 | 7,035 | 3,675 |

Abbreviations: Development, M: development data set for male; Development, F: development data set for female; Validation, M: validation data set for male; Validation, F: validation data set for female

**Table S3. Independent effects of chronological age and DAnHI on the risk of mortality.**

|  |  |  | Total |  | Non-cancer |  | Cancer |  |
| --- | --- | --- | --- | --- | --- | --- | --- | --- |
| Sex | Age range | Variable | HR (95%CIs) | P value | HR (95%CIs) | P value | HR (95%CIs) | P value |
| Male | ≤29 | CA | 1.177 (1.067–1.299) | 0.001 | 1.085 (0.954–1.234) | 0.214 | 1.219 (1.060–1.402) | 5.37 x 10^-03^ |
|  |  | DAnHI | 1.027 (0.993–1.061) | 0.121 | 1.042 (0.999–1.087) | 0.057 | 1.002 (0.954–1.053) | 0.934 |
|  | 30–39 | CA | 1.087 (1.063–1.111) | 2.01 x 10^-13^ | 1.045 (1.013–1.078) | 5.59 x 10^-03^ | 1.124 (1.090–1.159) | 9.86 x 10^-14^ |
|  |  | DAnHI | 1.070 (1.056–1.083) | 7.40 x 10^-26^ | 1.089 (1.070–1.107) | 5.38 x 10^-23^ | 1.052 (1.033–1.071) | 3.85 x 10^-08^ |
|  | 40–49 | CA | 1.105 (1.092–1.119) | 1.80 x 10^-55^ | 1.072 (1.052–1.092) | 3.59 x 10^-13^ | 1.132 (1.113–1.151) | 2.95 x 10^-48^ |
|  |  | DAnHI | 1.097 (1.091–1.104) | 8.10 x 10^-218^ | 1.131 (1.122–1.140) | 2.58 x 10^-215^ | 1.067 (1.058–1.076) | 9.98 x 10^-50^ |
|  | 50–59 | CA | 1.101 (1.091–1.111) | 4.36 x 10^-91^ | 1.084 (1.068–1.101) | 2.91 x 10^-25^ | 1.113 (1.100–1.126) | 2.38 x 10^-71^ |
|  |  | DAnHI | 1.086 (1.082–1.090) | <1.00 x 10^-310^ | 1.114 (1.108–1.120) | <1.00 x 10^-310^ | 1.069 (1.063–1.074) | 1.53 x 10^-149^ |
|  | 60–69 | CA | 1.114 (1.106–1.123) | 9.83 x 10^-175^ | 1.139 (1.125–1.152) | 2.30 x 10^-98^ | 1.104 (1.093–1.114) | 2.20 x 10^-90^ |
|  |  | DAnHI | 1.058 (1.055–1.061) | 6.73 x 10^-306^ | 1.071 (1.066–1.076) | 7.75 x 10^-191^ | 1.053 (1.049–1.057) | 8.67 x 10^-149^ |
|  | ≥70 | CA | 1.251 (1.239–1.263) | <1.00 x 10^-310^ | 1.356 (1.337–1.375) | <1.00 x 10^-310^ | 1.189 (1.172–1.206) | 4.21 x 10^-123^ |
|  |  | DAnHI | 1.035 (1.032–1.037) | 3.29 x 10^-194^ | 1.040 (1.037–1.043) | 1.36 x 10^-154^ | 1.034 (1.030–1.038) | 9.34 x 10^-77^ |
|  | Total | CA | 1.120 (1.119–1.122) | <1.00 x 10^-310^ | 1.134 (1.132–1.136) | <1.00 x 10^-310^ | 1.115 (1.114–1.117) | <1.00 x 10^-310^ |
|  |  | DAnHI | 1.055 (1.053–1.056) | <1.00 x 10^-310^ | 1.066 (1.064–1.069) | <1.00 x 10^-310^ | 1.049 (1.047–1.051) | <1.00 x 10^-310^ |
| Female | ≤29 | CA | 1.188 (1.077–1.311) | 6.02 x 10^-4^ | 1.140 (0.939–1.384) | 0.185 | 1.205 (1.076–1.350) | 1.23 x 10^-03^ |
|  |  | DAnHI | 1.042 (1.016–1.069) | 0.002 | 1.076 (1.031–1.123) | 7.83 x 10^-04^ | 1.023 (0.991–1.057) | 0.159 |
|  | 30–39 | CA | 1.079 (1.040–1.120) | 6.33 x 10^-5^ | 1.071 (0.996–1.152) | 0.063 | 1.080 (1.034–1.127) | 4.44 x 10^-04^ |
|  |  | DAnHI | 1.030 (1.011–1.050) | 0.002 | 1.050 (1.014–1.087) | 5.98 x 10^-03^ | 1.021 (0.999–1.044) | 0.064 |
|  | 40–49 | CA | 1.050 (1.034–1.067) | 1.44 x 10^-9^ | 1.021 (0.989–1.055) | 0.204 | 1.059 (1.040–1.078) | 6.55 x 10^-10^ |
|  |  | DAnHI | 1.041 (1.033–1.049) | 8.34 x 10^-24^ | 1.085 (1.070–1.101) | 1.73 x 10^-29^ | 1.026 (1.016–1.035) | 4.60 x 10^-08^ |
|  | 50–59 | CA | 1.093 (1.078–1.108) | 1.16 x 10^-35^ | 1.131 (1.101–1.162) | 9.67 x 10^-19^ | 1.082 (1.065–1.100) | 1.14 x 10^-21^ |
|  |  | DAnHI | 1.026 (1.020–1.032) | 1.61 x 10^-16^ | 1.054 (1.042–1.066) | 7.14 x 10^-20^ | 1.015 (1.008–1.023) | 3.58 x 10^-05^ |
|  | 60–69 | CA | 1.151 (1.138–1.165) | 1.35 x 10^-128^ | 1.219 (1.197–1.242) | 2.27 x 10^-99^ | 1.111 (1.095–1.128) | 5.40 x10^-45^ |
|  |  | DAnHI | 1.020 (1.015–1.025) | 9.60 x 10^-16^ | 1.032 (1.025–1.040) | 1.41 x 10^-17^ | 1.011 (1.005–1.018) | 6.21 x 10^-04^ |
|  | ≥70 | CA | 1.424 (1.405–1.443) | <1.00 x 10^-310^ | 1.565 (1.538–1.592) | <1.00 x 10^-310^ | 1.247 (1.221–1.273) | 1.28 x 10^-96^ |
|  |  | DAnHI | 1.008 (1.005–1.010) | 7.37 x 10^-9^ | 1.009 (1.006–1.013) | 3.77 x 10^-09^ | 1.005 (1.000–1.010) | 6.90 x 10^-02^ |
|  | Total | CA | 1.140 (1.138–1.142) | <1.00 x 10^-310^ | 1.215 (1.211–1.219) | <1.00 x 10^-310^ | 1.098 (1.095–1.100) | <1.00 x 10^-310^ |
|  |  | DAnHI | 1.014 (1.012–1.016) | 5.61 x 10^-39^ | 1.018 (1.015–1.020) | 7.63 x 10^-35^ | 1.011 (1.007–1.014) | 1.02 x 10^-10^ |

CA and DAnHI do not cause multicollinearity in the multivariable cox regression models: variance inflation factors (VIF) for CA and DAnHI are all less than 1.03 for both sexes and all age groups.

Abbreviations: Total: event is total mortality, Non-cancer: event is non-cancer death, Cancer: event is cancer death, CA: chronological age, DAnHI: differential aging health index, HR (95%CIs): hazard ratio and 95% confidence intervals.

**Table S4. Summary of mortality in the development and validation data set separated by the cause of death, sex, and broad age groups.**

| Age group | Type | Development, M | Development, F | Validation, M | Validation, F |
| --- | --- | --- | --- | --- | --- |
| ≤39 | Total participant | 485,359 | 329,831 | 208,387 | 140,582 |
|  | All death | 1,185 | 496 | 496 | 217 |
|  | Non-cancer | 593 | 129 | 259 | 54 |
|  | Cancer | 592 | 367 | 237 | 163 |
| 40–64 | Total participant | 543,664 | 657,327 | 232,340 | 282,429 |
|  | All death | 12,693 | 6,033 | 5,576 | 2,599 |
|  | Non-cancer | 4,912 | 1,667 | 2,136 | 759 |
|  | Cancer | 7,781 | 4,366 | 3,440 | 1,840 |
| ≥65 | Total participant | 76,502 | 95,472 | 33,070 | 40,973 |
|  | All death | 17,388 | 11,449 | 7,408 | 4,975 |
|  | Non-cancer | 9,311 | 7,636 | 4,050 | 3,303 |
|  | Cancer | 8,077 | 3,813 | 3,358 | 1,672 |
| Total | Total participant | 1,105,525 | 1,082,630 | 473,797 | 463,984 |
|  | All death | 31,266 | 17,978 | 13,480 | 7,791 |
|  | Non-cancer | 14,816 | 9,432 | 6,445 | 4,116 |
|  | Cancer | 16,450 | 8,546 | 7,035 | 3,675 |

Abbreviations: Development, M: development data set for male; Development, F: development data set for female; Validation, M: validation data set for male; Validation, F: validation data set for female

**Table S5. Prediction accuracy of the models, including CA and DAnHI, for predicting 10-year total mortality.**

| Gender | Age range | Statistics | DAnHI | CA | CA with DAnHI | BA |
| --- | --- | --- | --- | --- | --- | --- |
| Male | Total | AUC (95% CIs) | 0.777 (0.772–0.781) | 0.881 (0.878–0.884) | 0.888 (0.885–0.891) | 0.887 (0.884–0.890) |
|  |  | DeLong's P | <1.00 x 10^-310^ | Ref. | 2.86 x 10^-83^ | 4.59 x 10^-21^ |
|  | ≤39 | AUC (95% CIs) | 0.581 (0.554–0.608) | 0.638 (0.614–0.663) | 0.650 (0.625–0.674) | 0.643 (0.619–0.668) |
|  |  | DeLong's P | 6.30 x 10^-04^ | Ref. | 0.070 | 0.562 |
|  | 40–64 | AUC (95% CIs) | 0.663 (0.655–0.671) | 0.716 (0.709–0.722) | 0.745 (0.738–0.752) | 0.744 (0.737–0.751) |
|  |  | DeLong's P | 6.55 x 10^-28^ | Ref. | 4.20 x 10^-52^ | 1.30 x 10^-37^ |
|  | ≥65 | AUC (95% CIs) | 0.620 (0.612–0.628) | 0.693 (0.686–0.700) | 0.711 (0.705–0.718) | 0.681 (0.674–0.688) |
|  |  | DeLong's P | 3.34 x 10^-49^ | Ref. | 1.75 x 10^-33^ | 6.58 x 10^-04^ |
| Female | Total | AUC (95% CIs) | 0.748 (0.743–0.754) | 0.868 (0.864–0.873) | 0.869 (0.865–0.874) | 0.860 (0.855–0.864) |
|  |  | DeLong's P | <1.00 x 10^-310^ | Ref. | 2.75 x 10^-07^ | 8.19 x 10^-28^ |
|  | ≤39 | AUC (95% CIs) | 0.611 (0.572–0.649) | 0.676 (0.643–0.708) | 0.688 (0.655–0.720) | 0.680 (0.647–0.714) |
|  |  | DeLong's P | 5.23 x 10^-03^ | Ref. | 0.155 | 0.721 |
|  | 40–64 | AUC (95% CIs) | 0.604 (0.593–0.616) | 0.672 (0.661–0.683) | 0.677 (0.667–0.688) | 0.669 (0.658–0.680) |
|  |  | DeLong's P | 6.74 x 10^-27^ | Ref. | 1.59 x 10^-04^ | 0.272 |
|  | ≥65 | AUC (95% CIs) | 0.555 (0.545–0.564) | 0.731 (0.724–0.739) | 0.735 (0.727–0.742) | 0.642 (0.634–0.651) |
|  |  | DeLong's P | 1.62 x 10^-193^ | Ref. | 9.33 x 10^-05^ | 1.98 x 10^-92^ |

DAnHI: cox-model with differential aging and health index as a single risk factor. CA: cox-model with chronological age as a single risk factor. CA with DAnHI: cox-model with CA and DAnHI as complementary risk factors. BA: cox-model with biological age, defined as the sum of CA and DAnHI, as a single risk factor. AUC (95% CIs): area under the curve with 95% confidence intervals. DeLong's P: P-value computed by DeLong's method to compare AUCs between models.

Abbreviations: AUC: area under the curve, BA: biological age, CA: chronological age, Ref.: reference, DAnHI: differential aging and health index.

**Table S6. Prediction accuracy of the DAnHI-including models for predicting 10-year cancer death.**

| Sex | Age range | Statistics | DAnHI | CA | CA with DAnHI | BA |
| --- | --- | --- | --- | --- | --- | --- |
| Male | Total | AUC (95% CIs) | 0.753 (0.747–0.759) | 0.873 (0.869–0.877) | 0.877 (0.873–0.881) | 0.874 (0.870–0.878) |
|  |  | DeLong's P | <1.00 x 10^-310^ | Ref. | 1.53 x 10^-27^ | 0.062 |
|  | ≤39 | AUC (95% CIs) | 0.552 (0.515–0.589) | 0.641 (0.607–0.676) | 0.643 (0.608–0.677) | 0.625 (0.591–0.659) |
|  |  | DeLong's P | 1.82 x 10^-04^ | Ref. | 0.649 | 0.151 |
|  | 40–64 | AUC (95% CIs) | 0.641 (0.631–0.651) | 0.730 (0.722–0.739) | 0.745 (0.737–0.753) | 0.740 (0.732–0.748) |
|  |  | DeLong's P | 1.30 x 10^-51^ | Ref. | 1.39 x 10^-18^ | 1.09 x 10^-04^ |
|  | ≥65 | AUC (95% CIs) | 0.601 (0.590–0.611) | 0.643 (0.633–0.654) | 0.663 (0.653–0.673) | 0.645 (0.635–0.655) |
|  |  | DeLong's P | 1.84 x 10^-09^ | Ref. | 2.49 x 10^-14^ | 0.752 |
| Female | Total | AUC (95% CIs) | 0.708 (0.700–0.716) | 0.806 (0.798–0.813) | 0.807 (0.799–0.814) | 0.799 (0.792–0.806) |
|  |  | DeLong's P | 8.15 x 10^-139^ | Ref. | 3.04 x 10^-03^ | 2.76 x 10^-07^ |
|  | ≤39 | AUC (95% CIs) | 0.582 (0.538–0.626) | 0.652 (0.614–0.690) | 0.658 (0.620–0.696) | 0.647 (0.608–0.686) |
|  |  | DeLong's P | 0.011 | Ref. | 0.479 | 0.757 |
|  | 40~64 | AUC (95% CIs) | 0.588 (0.574–0.601) | 0.657 (0.644–0.670) | 0.660 (0.647–0.672) | 0.649 (0.636–0.662) |
|  |  | DeLong's P | 8.59 x 10^-21^ | Ref. | 0.025 | 0.036 |
|  | ≥65 | AUC (95% CIs) | 0.550 (0.535–0.565) | 0.644 (0.630–0.658) | 0.647 (0.633–0.662) | 0.602 (0.588–0.617) |
|  |  | DeLong's P | 1.59 x 10^-21^ | Ref. | 0.055 | 7.33 x 10^-09^ |

DAnHI: cox-model with differential aging and health index as a single risk factor. CA: cox-model with chronological age as a single risk factor. CA with DAnHI: cox-model with CA and DAnHI as complementary risk factors. BA: cox-model with biological age, defined as the sum of CA and DAnHI, as a single risk factor. AUC (95% CIs): area under the curve with 95% confidence intervals. DeLong's P: P-value computed by DeLong's method to compare AUCs between models.

Abbreviations: AUC: area under the curve, BA: biological age, CA: chronological age, Ref.: reference, DAnHI: differential aging and health index.

**Table S7. Comparison of prediction performance between chronological age and other biological age estimation algorithms in male cohort.**

| Mortality type | Model | AUC (95% CIs) | Sensitivity | Specificity | PPV | NPV | R^2^ | F1-score |
| --- | --- | --- | --- | --- | --- | --- | --- | --- |
| All death | Chronological age | 0.823 (0.818 - 0.828) | 0.729 | 0.767 | 0.735 | 0.761 | Ref. | 0.732 |
|  | DAnHI | 0.830 (0.825 - 0.835) | 0.747 | 0.766 | 0.739 | 0.774 | 0.897 | 0.743 |
|  | MLR | 0.724 (0.718 - 0.730) | 0.705 | 0.624 | 0.624 | 0.704 | 0.244 | 0.662 |
|  | PCA | 0.737 (0.731 - 0.743) | 0.773 | 0.592 | 0.627 | 0.746 | 0.483 | 0.692 |
|  | SGD | 0.687 (0.681 - 0.694) | 0.676 | 0.597 | 0.598 | 0.675 | 0.165 | 0.635 |
|  | DNN | 0.733 (0.727 - 0.739) | 0.645 | 0.702 | 0.657 | 0.69 | 0.330 | 0.651 |
|  | RF | 0.708 (0.702 - 0.715) | 0.641 | 0.665 | 0.629 | 0.676 | 0.282 | 0.635 |
| Death by non-cancer | Chronological age | 0.847 (0.841 - 0.853) | 0.699 | 0.856 | 0.677 | 0.868 | Ref. | 0.688 |
|  | DAnHI | 0.860 (0.854 - 0.866) | 0.739 | 0.842 | 0.668 | 0.882 | 0.906 | 0.702 |
|  | MLR | 0.758 (0.751 - 0.765) | 0.675 | 0.706 | 0.497 | 0.834 | 0.260 | 0.572 |
|  | PCA | 0.758 (0.752 - 0.765) | 0.798 | 0.603 | 0.464 | 0.874 | 0.509 | 0.587 |
|  | SGD | 0.713 (0.705 - 0.721) | 0.687 | 0.626 | 0.442 | 0.822 | 0.179 | 0.538 |
|  | DNN | 0.762 (0.755 - 0.770) | 0.686 | 0.709 | 0.505 | 0.839 | 0.351 | 0.582 |
|  | RF | 0.744 (0.736 - 0.751) | 0.686 | 0.675 | 0.477 | 0.833 | 0.306 | 0.563 |
| Death by cancer | Chronological age | 0.823 (0.817 - 0.829) | 0.748 | 0.744 | 0.588 | 0.858 | Ref. | 0.658 |
|  | DAnHI | 0.825 (0.819 - 0.831) | 0.742 | 0.753 | 0.595 | 0.857 | 0.906 | 0.660 |
|  | MLR | 0.708 (0.701 - 0.716) | 0.671 | 0.637 | 0.475 | 0.798 | 0.247 | 0.556 |
|  | PCA | 0.735 (0.728 - 0.742) | 0.781 | 0.575 | 0.474 | 0.843 | 0.522 | 0.590 |
|  | SGD | 0.676 (0.669 - 0.684) | 0.657 | 0.601 | 0.446 | 0.782 | 0.184 | 0.531 |
|  | DNN | 0.720 (0.713 - 0.728) | 0.609 | 0.716 | 0.511 | 0.789 | 0.327 | 0.556 |
|  | RF | 0.689 (0.681 - 0.696) | 0.599 | 0.676 | 0.475 | 0.775 | 0.267 | 0.530 |
|  |  |  |  |  |  |  |  |  |

From the validation data set, all mortality data and randomly selected alive data (sample ratio is 1:1) were used to analyze the prediction performance. Model: statistical or machine learning-based algorithms used to estimate biological age. DAnHI: differential aging and health index
MLR: multiple linear regression. PCA: principal component analysis. SGD: stochastic gradient descent. DNN: deep neural network.
RF: random forest. AUC (95% CIs): area under the curve and 95% confidence intervals. PPV: positive predictive value. NPV: negative predictive value. R^2^: coefficient of determination. Ref. : reference used to compute R^2^.

**Table S8. Comparison of prediction performance between chronological age and other biological age estimation algorithms in female cohort.**

| Mortality type | Model | AUC (95% CIs) | Sensitivity | Specificity | PPV | NPV | R^2^ | F1-score |
| --- | --- | --- | --- | --- | --- | --- | --- | --- |
| All death | Chronological age | 0.842 (0.836 - 0.849) | 0.767 | 0.779 | 0.753 | 0.792 | Ref. | 0.760 |
|  | DAnHI | 0.833 (0.827 - 0.840) | 0.759 | 0.777 | 0.749 | 0.786 | 0.913 | 0.754 |
|  | MLR | 0.713 (0.705 - 0.721) | 0.713 | 0.606 | 0.613 | 0.707 | 0.314 | 0.659 |
|  | PCA | 0.760 (0.753 - 0.768) | 0.771 | 0.641 | 0.654 | 0.761 | 0.544 | 0.708 |
|  | SGD | 0.715 (0.707 - 0.723) | 0.701 | 0.621 | 0.619 | 0.703 | 0.313 | 0.657 |
|  | DNN | 0.726 (0.719 - 0.734) | 0.684 | 0.649 | 0.631 | 0.701 | 0.433 | 0.656 |
|  | RF | 0.710 (0.702 - 0.718) | 0.658 | 0.649 | 0.622 | 0.684 | 0.387 | 0.639 |
| Death by non-cancer | Chronological age | 0.911 (0.905 - 0.916) | 0.821 | 0.866 | 0.558 | 0.72 | Ref. | 0.664 |
|  | DAnHI | 0.900 (0.895 - 0.906) | 0.868 | 0.809 | 0.681 | 0.929 | 0.920 | 0.763 |
|  | MLR | 0.755 (0.746 - 0.764) | 0.772 | 0.614 | 0.485 | 0.851 | 0.329 | 0.596 |
|  | PCA | 0.807 (0.800 - 0.815) | 0.836 | 0.659 | 0.536 | 0.895 | 0.568 | 0.653 |
|  | SGD | 0.759 (0.750 - 0.768) | 0.724 | 0.667 | 0.506 | 0.837 | 0.331 | 0.596 |
|  | DNN | 0.769 (0.760 - 0.778) | 0.740 | 0.658 | 0.504 | 0.843 | 0.452 | 0.600 |
|  | RF | 0.753 (0.743 - 0.762) | 0.692 | 0.675 | 0.501 | 0.823 | 0.409 | 0.581 |
| Death by cancer | Chronological age | 0.798 (0.790 - 0.807) | 0.669 | 0.783 | 0.578 | 0.842 | Ref. | 0.620 |
|  | DAnHI | 0.790 (0.781 - 0.798) | 0.699 | 0.745 | 0.548 | 0.848 | 0.914 | 0.614 |
|  | MLR | 0.686 (0.676 - 0.696) | 0.640 | 0.635 | 0.437 | 0.799 | 0.313 | 0.519 |
|  | PCA | 0.732 (0.722 - 0.741) | 0.695 | 0.665 | 0.479 | 0.831 | 0.561 | 0.567 |
|  | SGD | 0.687 (0.676 - 0.697) | 0.624 | 0.652 | 0.443 | 0.796 | 0.312 | 0.518 |
|  | DNN | 0.699 (0.689 - 0.710) | 0.627 | 0.667 | 0.454 | 0.801 | 0.437 | 0.527 |
|  | RF | 0.681 (0.671 - 0.691) | 0.597 | 0.672 | 0.447 | 0.79 | 0.383 | 0.511 |
|  |  |  |  |  |  |  |  |  |

From the validation data set, all mortality data and randomly selected alive data (sample ratio is 1:1) were used to analyze the prediction performance. Model: statistical or machine learning-based algorithms used to estimate biological age. DAnHI: differential aging and health index. MLR: multiple linear regression. PCA: principal component analysis. SGD: stochastic gradient descent. DNN: deep neural network. RF: random forest. AUC (95% CIs): area under the curve and 95% confidence intervals. PPV: positive predictive value. NPV: negative predictive value. R^2^: coefficient of determination. Ref. : reference used to compute R^2^.
